# Pancreas and duodenum herniation in a giant inguinal hernia sac in a patient with severe scoliosis: a rare case report

**DOI:** 10.1093/jscr/rjaf471

**Published:** 2025-07-03

**Authors:** Radomir Gelevski, Gjorgji Jota, Gjorgji Trajkovski, Andrej Nikolovski, Vladimir Andreevski, Gjorgji Deriban

**Affiliations:** Department of Surgery, General Hospital Kumanovo, Kumanovo 1300 North Macedonia; Ss. Cyril and Methodius University, University Clinic for Digestive Surgery, Skopje 1000, North Macedonia; Ss. Cyril and Methodius University, Medical Faculty, Skopje 1000, North Macedonia; Ss. Cyril and Methodius University, Medical Faculty, Skopje 1000, North Macedonia; Department of Visceral Surgery, University Surgery Hospital “Sv. Naum Ohridski”, Skopje 1000, North Macedonia; Ss. Cyril and Methodius University, University Clinic for Gastroenterohepathology, Skopje 1000, North Macedonia; Ss. Cyril and Methodius University, University Clinic for Gastroenterohepathology, Skopje 1000, North Macedonia

**Keywords:** giant inguinal hernia, pancreas, duodenum, scoliosis

## Abstract

Giant inguinal hernias (GIHs) are rare clinical entities, typically containing omentum or small bowel. Involvement of retroperitoneal organs, such as the pancreas and duodenum, is exceedingly uncommon due to their fixed anatomical positions. We report a unique case of a 52-year-old male with a longstanding right GIH and severe scoliosis, in whom preoperative imaging and surgical exploration revealed herniation of the pancreatic head and duodenum into the hernia sac. Contributing factors included altered retroperitoneal geometry from spinal curvature, reduced abdominal wall tone, and congenital right hip displacement with associated functional limitation. Incidental findings of multiple left hepatic duct calculi raised concerns for biliary stasis due to chronic duodenal displacement. This case highlights the importance of considering atypical hernia content in patients with longstanding hernias and complex musculoskeletal deformities, and underscores the role of comprehensive imaging and multidisciplinary assessment in surgical planning.

## Introduction

Inguinal hernias are among the most common conditions encountered in general surgery, typically involving small bowel or omentum. However, a subset of patients presents with giant inguinal hernias (GIHs), defined as hernias extending below the midpoint of the thigh in a standing position [1]. These cases are rare and often result from prolonged neglect or lack of access to surgical care.

The size and chronicity of GIHs increase the likelihood of containing atypical organs. Nonetheless, retroperitoneal organs, including the pancreas and duodenum, are extremely rarely involved [2]. Their anatomical position and fixation within the posterior abdominal wall usually prevent such displacement. Only a handful of cases in literature have documented herniation of these structures, typically through other abdominal wall defects or as incidental findings during surgery [3].

Scoliosis, particularly of the lumbar spine, introduces additional complexity by altering the normal configuration and pressure dynamics of the abdominal cavity [4]. This can potentially contribute to atypical herniation patterns due to distortion of fascial planes and shifting of intra-abdominal contents. However, the relationship between scoliosis and retroperitoneal organ herniation has not been widely explored.

Moreover, long-standing hernias can result in progressive enlargement of the hernia sac and stretching of the abdominal wall, reducing its ability to retain intra-abdominal organs. In these cases, structures that are typically considered fixed, such as the pancreas and duodenum, may become mobile enough to protrude through anatomical defects under sustained pressure [5].

In this report, we present what appears to be the first documented case of herniation of the pancreatic head and duodenum into a GIH sac in the context of severe scoliosis, highlighting the importance of comprehensive imaging and multidisciplinary management in such rare and challenging presentations.

## Case report

A 52-year-old male presented with a massive, irreducible right inguinoscrotal hernia present for over two decades. The hernia had progressively enlarged and was associated with abdominal discomfort and postprandial bloating. The patient also had a history of untreated severe lumbar scoliosis and congenital right hip displacement, which further contributed to asymmetry and altered abdominal dynamics.

Physical examination revealed a large, non-tender, irreducible hernia extending to mid-thigh level. Abdominal examination showed mild scaphoid configuration with decreased bowel sounds over the hernia. The patient had an antalgic gait due to hip dysplasia. Laboratory results were within normal limits.

**Figure 1:**
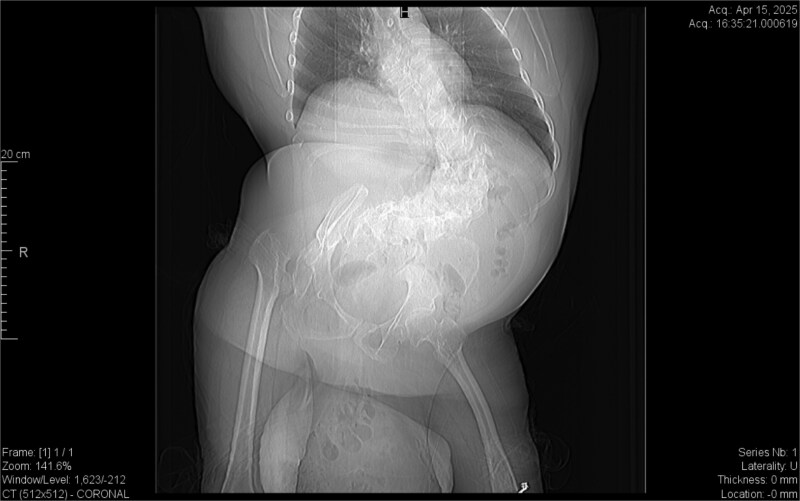
Dextroscoliosis with marked rotation of vertebral bodies, displaced right femoral head and giant inguinal hernia.

From the provided coronal CT image (Fig. 1), the following findings are noted: The spinal curvature is prominently right-convex in the lumbar region, indicating a dextroscoliosis with marked rotation of vertebral bodies, causing asymmetrical displacement of the retroperitoneal organs. The curvature shortens the abdominal cavity vertically on the left side, while expanding the retroperitoneal volume on the right. The right femoral head is elevated and laterally displaced, consistent with developmental dysplasia of the hip. There is flattening of the acetabulum and apparent false articulation with the iliac wing. These findings suggest chronic alteration of pelvic symmetry with pelvic tilt and subsequent uneven distribution of abdominal and intra-pelvic pressure. The combined effects of spinal curvature and pelvic deformity create an aberrant vector of force and organ positioning, contributing to the downward displacement of the pancreas and duodenum.

**Figure 2:**
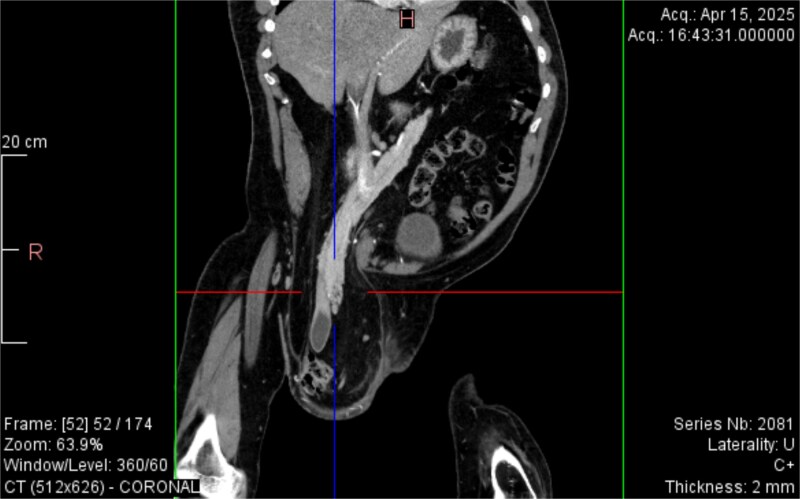
Coronal CT view inguinal hernial sac with pancreatic head and D2 and D3 segment of duodenum.

**Figure 3:**
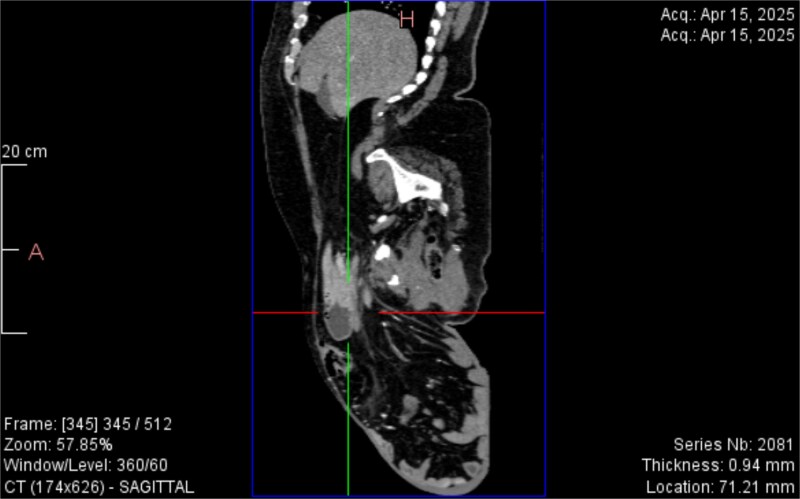
Sagittal CT view inguinal hernial sac with pancreatic head and D2 and D3 segment of duodenum.

**Figure 4:**
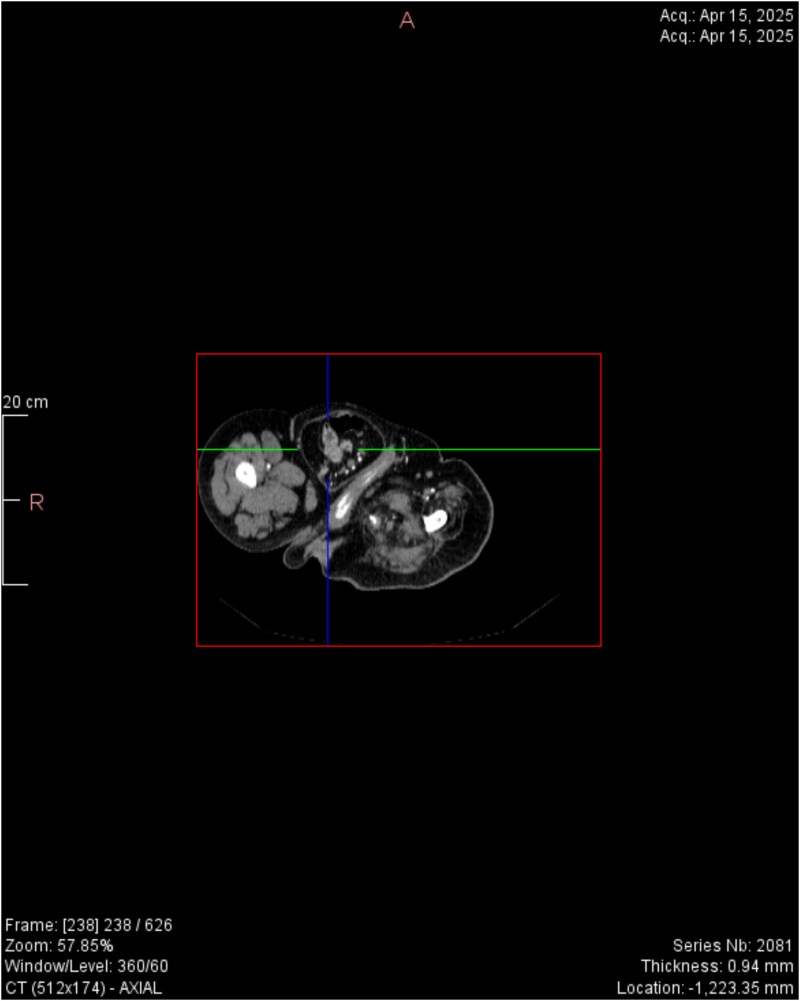
Horizontal CT view inguinal hernial sac with pancreatic head and D2 and D3 segment of duodenum.

**Figure 5:**
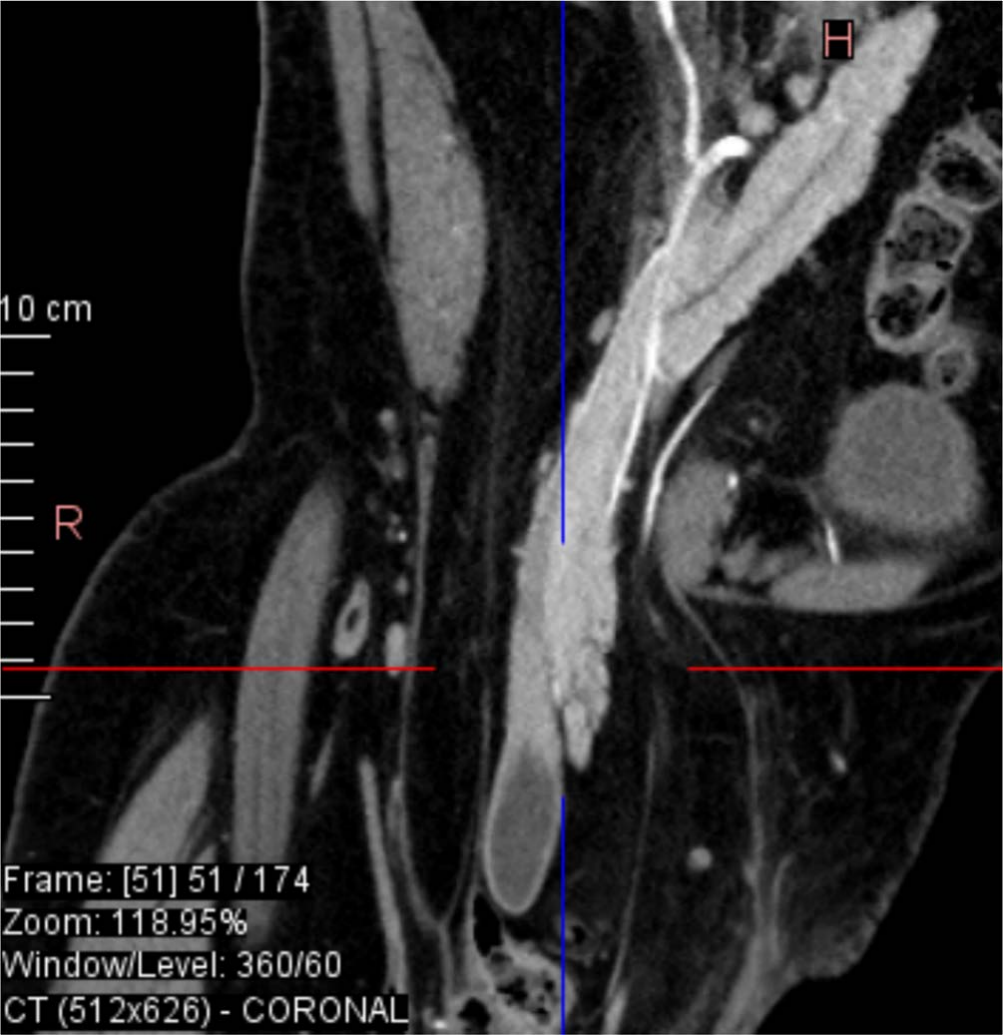
Coronal CT view visualization of vascularization of pancreatic head after i.v. contrast.

CT imaging (Figs 2–5) revealed a right inguinal hernia containing small bowel loops, ascending colon, the second and third portions of the duodenum, and the pancreatic head. The pancreatic head appeared rotated and displaced inferiorly into the hernia sac, without signs of acute pancreatitis.

Additionally, incidental findings (Fig. 2) included multiple calculi within the left hepatic duct. Though the patient was asymptomatic from a hepatobiliary standpoint, the presence of these stones raised concern for potential biliary stasis.

Functional assessments revealed limited range of motion in the right hip joint, with hip abduction restricted to 10° and flexion limited to 45°. Gait analysis demonstrated an antalgic pattern with decreased right stride length and compensatory pelvic tilt. The Timed Up and Go (TUG) test result was 18.2 s, indicating moderately impaired mobility. The Barthel Index for activities of daily living was 65/100, suggesting moderate dependence.

## Discussion

This case highlights a rare presentation of GIH involving retroperitoneal organ herniation, facilitated by altered anatomical positioning due to scoliosis. The displacement of the pancreas and duodenum is an exceptional finding, likely enabled by the chronic nature of the hernia and mechanical distortion of intra-abdominal structures.

In particular, spinal curvature in severe scoliosis may result in significant alterations to retroperitoneal geometry. The abnormal vertebral alignment can displace the posterior abdominal wall and compress or elongate adjacent fascial planes, which in turn may lead to a downward and lateral shift of retroperitoneal organs. These shifts can change the mechanical vectors acting upon structures like the duodenum and pancreas, increasing their susceptibility to gravitational traction and herniation—especially when the abdominal wall is weakened, as seen in GIH. The concave side of the spinal curvature may act as a ‘path of least resistance,’ directing organ displacement toward areas of reduced pressure or structural support.

Furthermore, the patient’s congenital right hip displacement likely contributed to long-term asymmetry in pelvic alignment and restricted mobility, which may have exacerbated postural adaptations and muscular imbalances. Reduced mobility can indirectly increase intra-abdominal pressure due to physical inactivity and altered biomechanics, potentially accelerating hernia progression and compounding the effects of spinal deformity.

The presence of multiple calculi within the left hepatic duct may reflect biliary stasis potentially linked to abnormal organ positioning or subclinical external compression. It is plausible that chronic duodenal displacement altered the angle or patency of the biliary tree, promoting cholestasis and the formation of intrahepatic calculi over time. Though the patient was asymptomatic, these findings suggest the need for vigilance in patients with complex abdominal and skeletal deformities, where organ malposition might contribute to secondary pathologies.

Thorough preoperative imaging and multidisciplinary planning are essential for managing such complex cases.

## Conclusion

Pancreatic and duodenal herniation into an inguinal hernia sac is extremely rare and may be facilitated by long-standing hernia and spinal deformity. Preoperative imaging, careful surgical planning, and multidisciplinary collaboration are key to successful outcomes in such unusual presentations.

## References

[1] Karthikeyan VS, Sistla SC, Ram D, et al. Giant inguinoscrotal hernia – report of a rare case with literature review. Int Surg 2014;99:560–4. 10.9738/INTSURG-D-13-00083.1 25216421 PMC4253924

[2] Ballard D, Sangster G. Pancreas-containing inguinal hernia in an adult with intestinal malrotation. Image Surg 2017;162:1185–6. 10.1016/j.surg.2016.12.025 28139243

[3] Gaedcke J, Schuler P, Brinker J, et al. Emergency repair of giant inguinoscrotal hernia in a septic patient. J Gastrointest Surg 2013;17:837–9. 10.1007/s11605-012-2136-7 23299222 PMC3599162

[4] Dericioglu BI, Ozgoren AO, Angin S. Adolescent idiopathic scoliosis causes pelvic flor dysfunction: a cross-sectional study. J Back Musculoskelet Rehabil 2025;38:314–23. 10.1177/10538127241301667 39973264

[5] Sewalia P, Chawla AS, Sangtam LT, et al. Repair of giant inguino-scrotal hernia with loss of domain using minimally invasive anterior component separation technique combined with Lichtenstein tension-free mesh hernioplasty. Int Surg J 2021;8:406–10. 10.18203/2349-2902.isj20205915

